# Increased arterial stiffness and atherosclerosis in patients with nonfunctioning adrenal incidentaloma

**DOI:** 10.1038/s41440-025-02398-3

**Published:** 2025-10-06

**Authors:** Mirko Parasiliti-Caprino, Elisa Febbraro, Bartolomeo Sangermano, Daniele Giuseppe Candela, Stefano Arata, Matteo Procopio, Martina Bollati, Chiara Lopez, Ezio Ghigo, Mauro Maccario

**Affiliations:** Arterial Hypertension and Cardiovascular Endocrinology Laboratory; Division of Endocrinology, Diabetes and Metabolism; City of Health and Science University Hospital; Department of Medical Sciences; University of Turin, Turin, Italy

**Keywords:** Pulse wave analysis, Pulse wave velocity, Adrenal adenoma, Carotid intima-media thickness, Cardiovascular risk, Implemental hypertension

## Abstract

Nonfunctioning adrenal incidentalomas (NFAIs) are typically considered benign and hormonally inactive, yet emerging evidence suggests a potential association with increased cardiovascular risk. This study aimed to evaluate whether NFAIs are independently associated with vascular damage, assessed through arterial stiffness and carotid Doppler ultrasound. In this observational study, we prospectively enrolled outpatients undergoing evaluation for suspected secondary hypertension at the Arterial Hypertension and Cardiovascular Endocrinology Laboratory of the University of Turin. Patients with NFAI were compared to individuals with essential hypertension and normal adrenal imaging. Cardiometabolic and hemodynamic parameters, including augmentation index (AIx), pulse wave velocity, carotid intima-media thickness, and plaque presence, were assessed. A total of 25 patients with NFAI and 165 controls were included. Univariate analysis revealed a worse cardiometabolic profile in NFAI patients. In multivariable models, NFAI was independently associated with increased arterial stiffness: AIx P2/P1 (EC 1.097, 95% CI 1.016–1.185; p = 0.019), AIx AP/PP (EC 1.133, 95% CI 1.043–1.232; p = 0.004), and AIx@HR75 (EC 1.073, 95% CI 1.004–1.147; p = 0.039). Pulse wave velocity was also significantly higher in the NFAI group (EC 2.144, 95% CI 1.021–4.502; p = 0.044). Furthermore, NFAI was associated with increased odds of having carotid intima-media thickness ≥0.9 mm (OR 3.312, 95% CI 1.011–10.855; p = 0.048), independent of other confounders. This is the first study to demonstrate that NFAI is independently associated with increased arterial stiffness and early vascular remodeling. These findings support the need for systematic cardiovascular risk assessment in patients with NFAI, even in the absence of overt hormonal activity.

**Figure:**
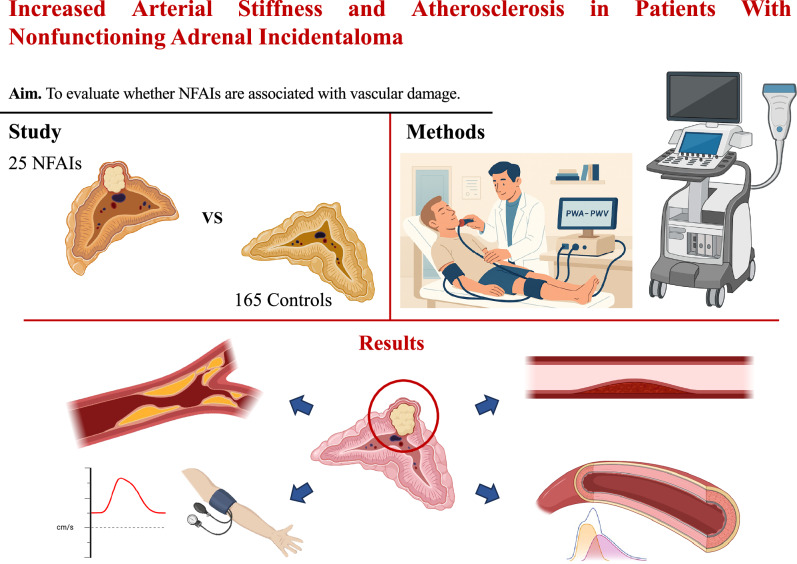
This graphical abstract illustrates the findings of a cross-sectional study comparing 25 patients with nonfunctioning adrenal incidentaloma (NFAI) to 165 controls with essential hypertension. All participants underwent a comprehensive cardiometabolic and vascular evaluation, including pulse wave analysis (PWA), pulse wave velocity (PWV), and carotid Doppler ultrasound. Compared to controls, patients with NFAI displayed increased arterial stiffness—demonstrated by higher augmentation index (AIx) and PWV values—as well as a higher prevalence of carotid intimamedia thickness (cIMT) ≥0.9 mm. These vascular alterations occurred despite the absence of overt hormonal hypersecretion. The results support the interpretation of NFAI as a potential marker of early vascular remodeling and advocate for systematic cardiovascular risk assessment in these patients

This graphical abstract illustrates the findings of a cross-sectional study comparing 25 patients with nonfunctioning adrenal incidentaloma (NFAI) to 165 controls with essential hypertension. All participants underwent a comprehensive cardiometabolic and vascular evaluation, including pulse wave analysis (PWA), pulse wave velocity (PWV), and carotid Doppler ultrasound. Compared to controls, patients with NFAI displayed increased arterial stiffness—demonstrated by higher augmentation index (AIx) and PWV values—as well as a higher prevalence of carotid intimamedia thickness (cIMT) ≥0.9 mm. These vascular alterations occurred despite the absence of overt hormonal hypersecretion. The results support the interpretation of NFAI as a potential marker of early vascular remodeling and advocate for systematic cardiovascular risk assessment in these patients

## Introduction

An adrenal incidentaloma (AI) is an adrenal mass ≥1 cm in diameter discovered incidentally during imaging not intended for adrenal evaluation [1]. Since the early 2000s, the detection rate of AI has markedly increased due to the wider availability and improved resolution of imaging techniques. Once an adrenal mass is identified, a thorough workup is warranted to distinguish benign from malignant lesions and functioning from nonfunctioning tumors.

It is widely accepted that 70–80% of these tumors are nonfunctioning adrenal incidentalomas (NFAIs) [2]: benign adrenal masses without clinical or mild hormone secretion. Although classically considered clinically inert, emerging evidence suggests that NFAIs may confer increased cardiometabolic risk compared to the general population [3–14], prompting the possibility to improve the clinical care of such patients. However, previous studies have been limited by small sample sizes, heterogeneous control groups, and inconsistent diagnostic criteria, leaving the true clinical significance of NFAI incompletely defined.

Arterial stiffness and carotid intima-media thickness (cIMT) are robust predictors of vascular and metabolic complications, allowing physicians to foresee possible comorbidities and start preventive therapy [15–17]. Patients with NFAI, who apparently are healthy, could instead have preclinical signs of vascular or metabolic alterations, not visible at physical or laboratory examination, but that could be found with instrumental exams and warn the physicians of possible risk of future events.

Existing data on arterial stiffness in NFAI patients are limited and primarily based on non–gold-standard methods [18, 19]. Similarly, studies assessing carotid structure have typically relied on basic cIMT measurements without comprehensive vascular imaging. Therefore, this study aimed to investigate, in a prospective cohort referred to a tertiary care center, whether NFAI is independently associated with increased cardiometabolic burden and vascular damage, as assessed by state-of-the-art arterial stiffness evaluation and detailed carotid Doppler ultrasound analysis.

## Methods

### Design and study population

This cross-sectional study was conducted in accordance with the STROBE guidelines for reporting observational research [20]. Consecutive adult patients referred to the Arterial Hypertension and Cardiovascular Endocrinology Laboratory of the Division of Endocrinology, Diabetes, and Metabolism (City of Health and Science University Hospital of Turin) between February 2023 and February 2025 for suspected secondary hypertension were evaluated.

Inclusion criteria were: age ≥18 years, and either a confirmed diagnosis of nonfunctioning adrenal incidentaloma (NFAI) or the documented absence of adrenal masses (by CT/MRI within the previous two years). Exclusion criteria were: a history of cardiovascular events or significant valvular disease, the presence of any functioning adrenocortical tumor, or other secondary forms of hypertension.

NFAI was defined by the following criteria: (1) diagnosis of an adrenal lesion of >10 mm discovered incidentally during abdominal imaging not performed for adrenal disease suspicion, (2) cortisol after dexamethasone suppression test <18 µg/L, (3) aldosterone-to-renin ratio <37 ng/mUI, without clinical or biochemical signs of mineralocorticoid excess, and (4) normal values of daily urinary (dU) or plasma metanephrines.

Clinical characteristics (weight, height, body mass index—BMI), demographic parameters (age and sex), family, pharmacological and medical history, office blood pressure (BP) values, laboratory data [fasting blood glucose, HbA1c, low-density lipoprotein (LDL), high-density lipoprotein (HDL), triglyceride, total cholesterol, creatinine, eGFR (estimated using the CKD-EPI formula), renin, aldosterone, cortisol after 1 mg-dexamethasone suppression test, urine and plasma metanephrine levels], were collected. Both cases and controls were assessed for the presence of type 2 diabetes mellitus (T2DM) and metabolic syndrome, by using NCEP ATP III criteria [21].

All patients provided written informed consent. The study was approved by the local Ethics Committee of the City of Health and Science University Hospital of Turin (Protocol No. 0034189) and conducted in accordance with the Declaration of Helsinki.

### Blood pressure measurements

Office BP was measured according to current guidelines [22]. BP control was defined as an average office BP less than 140/90 mmHg.

All participants underwent 24-h ambulatory blood pressure monitoring (ABPM) using a validated, automated oscillometric device (TM-2430; Intermed S.r.l., Milan, Italy). Measurements were recorded every 15 min during the daytime and every 20 minutes during the night. A valid ABPM recording required ≥80% of measurements to be successful. Controlled ambulatory BP was defined according to contemporary diagnostic thresholds (24). Heart rate variability (HRV) was determined as the standard deviation of daytime, night-time and 24-h heart rate (HR) obtained by ABPM. Nocturnal BP patterns were classified as follows: (1) reverse dipping when night-time BP was higher than daytime one, (2) reduced dipping a night reduction of 0–10%, (3) normal dipping a night reduction of 10–20%, and (4) extreme dipping a night reduction of more than 20%. Ambulatory arterial stiffness index was determined according to a proposed formula [23].

### Arterial stiffness and carotid Doppler ultrasound evaluation

All vascular assessments, including pulse wave analysis (PWA), pulse wave velocity (PWV), and carotid Doppler ultrasound, were performed by the same trained physician (M.P.C.) who was blinded to the final diagnosis, in order to minimize inter-operator variability and diagnostic bias.

Wave reflection was quantified using the augmentation index (AIx), which was expressed in three forms: (a) the augmented pressure (AP) to pulse pressure (PP) ratio [AIx (AP/PP)], representing the proportion of systolic pressure attributed to the reflected wave; (b) the second systolic peak (P2) to first systolic peak (P1) ratio [AIx (P2/P1)], reflecting the relative magnitude of the reflected wave; (c) AIx@HR75, which normalizes AIx (AP/PP) to a heart rate of 75 bpm, accounting for intra-individual variability in wave reflection due to heart rate [24, 25].

Carotid Doppler ultrasound was performed using the MyLab XPRO80 system with an L3–11 linear probe (Esaote SpA, Genoa, Italy). The examination included both transverse and longitudinal scans of the supra-aortic vessels bilaterally. Three techniques were systematically employed: B-mode imaging, for vessel wall morphology and measurement of carotid intima-media thickness (cIMT) and plaque detection; color Doppler imaging, for assessment of flow direction, turbulence, or aliasing; and pulsed-wave Doppler, for determining peak systolic velocity (PSV), end-diastolic velocity (EDV), and internal carotid artery to common carotid artery (ICA/CCA) velocity ratio.cIMT was measured using the QIMT algorithm (Esaote SpA, Genoa, Italy) during longitudinal B-mode scanning of each common carotid artery. The region of interest was positioned 1–2 cm proximal to the carotid bifurcation along the far wall. The software automatically acquired eight cIMT measurements and calculated the mean and standard deviation [26].

### Analytical methods

All hormonal and biochemical measurements were performed at the Baldi and Riberi Laboratory of the City of Health and Science University Hospital of Turin.

Urinary (µg/day) and plasma (pmol/L) metanephrines were measured by liquid chromatography–tandem mass spectrometry (LC-MS/MS) using the MassChrom platform (Chromsystems Instruments & Chemicals GmbH, Gräfelfing, Germany). Analytical validation was performed on a SCIEX Triple Quad 4500 system (SCIEX, Danaher, Washington D.C., USA), with limits of quantification of 4 µg/L for both analytes. Intra- and inter-assay coefficients of variation (CVs) were 2.8% and 2.7% for normetanephrine, and 8.6% and 7.5% for metanephrine.

Serum aldosterone (pg/mL) and direct renin concentration (DRC; µIU/mL) were determined by chemiluminescent immunoassay (CLIA) using the LIAISON platform (DiaSorin Biomedica S.p.A., Saluggia, Italy). For aldosterone: analytical sensitivity was 1.91 ng/dL; intra- and inter-assay CVs ranged from 2.1–4.2% and 5.8–10.5%, respectively. For renin: sensitivity ranged from 0.52–0.97 µIU/mL; intra- and inter-assay CVs were 2.52–4.87% and 7.01–13.03%, respectively.

Serum cortisol (µg/dL) was measured by chemiluminescent microparticle immunoassay (CMIA) using the Architect platform (Abbott Ireland Diagnostics Division, Illinois, USA). The assay had an analytical sensitivity of 0.7 µg/dL, with intra- and inter-assay CVs of 3.4–4.3% and 4.2–5.1%, respectively.

All other biochemical variables were measured in serum, plasma, or urine using standardized laboratory protocols.

#### Statistical analysis

Baseline demographic, clinical, and instrumental data were summarized as means and standard deviation for continuous variables, and as percentages for categorical variables. Comparisons between patients with and without NFAI were conducted using the Student’s *t*-test for continuous variables and the chi-square or Fisher’s exact test for categorical variables, as appropriate.

Pairwise exploratory correlation analysis was performed to investigate the associations among clinical, biochemical, and vascular parameters. For each pairwise comparison: when both variables were continuous, Spearman’s rank correlation test was applied. When at least one variable was categorical, the Kruskal–Wallis test was used to assess differences in distribution.

The resulting p-values were organized into a correlation matrix and visualized as a hierarchically clustered heatmap using the clustermap() function from the Seaborn library (v0.11.2). Average linkage clustering was independently applied to both rows and columns, enabling the identification of variable clusters with similar correlation patterns. The heatmap was rendered using the reversed “magma” colormap, with yellow tones indicating stronger associations (lower p values) and black representing weaker or nonsignificant associations (higher p values). The analysis was conducted in Python 3.11 using Pandas (v2.1), NumPy (v1.26), SciPy (v1.11), Matplotlib (v3.8), and Seaborn.

The association between NFAI and increased cIMT ( ≥ 0.9 mm) was analyzed using multivariable logistic regression models that included established vascular risk factors as covariates. Associations between NFAI and continuous arterial stiffness parameters (PWA and PWV) were assessed using multivariable linear regression, adjusting for clinically relevant covariates that are not incorporated into the calculation of these indices.

All statistical tests were two-sided, and a p value < 0.05 was considered statistically significant. Analyses were performed using Stata 18.0 (StataCorp LLC, College Station, TX, USA).

## Results

**Fig. 1:**
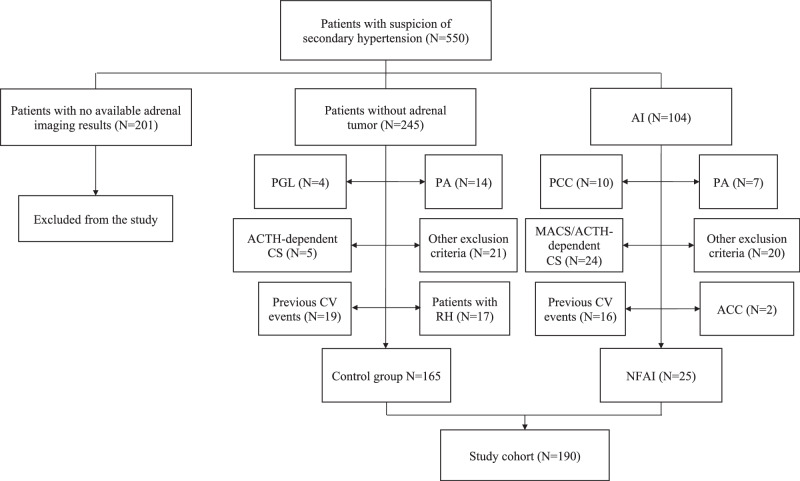
Flow-chart of the study. ACC adrenocortical carcinoma, AI adrenal incidentaloma, CV cardiovascular, PA primary aldosteronism, PCC pheochromocytoma, MACS mild autonomous cortisol secretion, NFAI nonfunctioning adrenal incidentaloma, RH resistant hypertension

After applying inclusion and exclusion criteria, 25 patients with NFAI (13 women [52%], 12 men [48%]) and 165 control subjects (104 women [63%], 61 men [37%]) were included in the analysis (Fig. 1).

Baseline characteristics of both groups are presented in Tables 1–3. Among patients with NFAI, the mean maximum lesion size was 22.2 ± 9.9 mm. Bilateral adrenal tumors were present in 20% of cases, while unilateral involvement was evenly distributed, with 40% of tumors located in the right adrenal gland and 40% in the left adrenal gland.

**Table 1:** Univariate analysis of clinical features and ABPM data between NFAI and control groups

Variables/Parameters		Total (N = 190)	Controls (N = 165)	NFAI (N = 25)	p-value
Age (years)		60.2 ± 15.6	59.4 ± 16.0	65.4 ± 11.5	0.074
Male Sex (%)		38.4	37.0	48.0	0.378
Hypertension Duration (years)		8.8 ± 9.8	8.4 ± 9.9	10.2 ± 10.0	0.497
FH of CVD (%)		17.6	18.5	13.0	0.530
Smoking Habit (%)		31.7	29.7	41.7	0.249
Weight (Kg)		74.3 ± 18.3	73.1 ± 17.8	80.1 ± 20.2	0.097
BMI (Kg/m^2^)		27.2 ± 5.3	26.8 ± 5.2	29.2 ± 5.5	0.055
SBP (mmHg)		135.1 ± 17.2	135.2 ± 17. 6	135.1 ± 16.2	0.977
DBP (mmHg)		81.4 ± 13.3	82.4 ± 13.5	76.9 ± 11.5	0.080
Sodium (mmol/L)		139.1 ± 2.8	139.0 ± 2.9	139.6 ± 2.3	0.381
Potassium (mmol/L)		4.3 ± 0.4	4.3 ± 0.4	4.3 ± 0.5	0.607
Glucose (mg/dL)		97.8 ± 26.1	98.0 ± 27.5	96.1 ± 17.1	0.742
HbA1c (mmol/mol)		38.7 ± 9.4	39.2 ± 9.6	36.3 ± 8.3	0.184
Total cholesterol (mg/dL)		195.0 ± 47.1	198.3 ± 47.2	175.8 ± 43.1	0.034
HDL-cholesterol (mg/dL)		57.5 ± 13.9	58.0 ± 14.4	54.6 ± 11.2	0.280
Triglycerides (mg/dL)		106.2 ± 50.5	107.3 ± 51.8	99.8 ± 42.8	0.514
LDL-cholesterol (mg/dL)		117.1 ± 40.5	120.1 ± 40.0	100.4 ± 40.4	0.028
Uric Acid (mg/dL)		5.4 ± 1.4	5.5 ± 1.3	5.2 ± 1.7	0.436
Corrected calcium (mmol/L)		2.4 ± 0.1	2.4 ± 0.1	2.4 ± 0.1	0.453
TSH (mIU/L)		1.6 ± 1.1	1.6 ± 1.0	1.8 ± 1.7	0.487
dU-normetanephrine (μg/day)		356.2 ± 157.7	342.1 ± 170.2	389.1 ± 121.0	0.257
dU-metanephrine (μg/day)		126.0 ± 62.8	117.9 ± 67.8	144.8 ± 45.2	0.102
P-normetanephrine (pmol/L)		725.8 ± 348.5	675.2 ± 308.3	827.0 ± 406.6	0.096
P-metanephrine (pmol/L)		173.6 ± 90.5	165.1 ± 99.4	190.6 ± 67.9	0.284
Renin (mUI/L)		12.4 ± 7.6	12.2 ± 7.3	14.2 ± 8.8	0.290
Aldosterone (ng/L)		252.1 ± 169.4	249.1 ± 173.2	276.1 ± 142.4	0.347
ARR (ng/mUI)		20.9 ± 8.0	20.8 ± 8.2	21.3 ± 7.3	0.774
Cortisol after 1 mg-DST (μg/L)		12.6 ± 3.8	12.2 ± 4.6	13.0 ± 2.9	0.496
No of antihypertensive drugs		1.7 ± 1.3	1.6 ± 1.2	1.8 ± 1.6	0.652
Antidyslipidemic drugs (%)		34.5	33.6	39.1	0.610
ACE inhibitors (%)		29.6	28.6	34.8	0.619
ARBs (%)		21.8	22.7	17.4	0.784
ACEi/ARBs (%)		51.4	51.3	52.2	1.000
CCB (%)		40.1	40.3	39.1	1.000
Thiazide (-like) diuretics (%)		27.5	27.7	26.1	1.000
MRAs (%)		7.0	7.6	4.3	1.000
β-blockers (%)		31.0	28.6	43.5	0.217
α-blockers (%)		6.3	4.2	17.4	0.038
α2-agonists (%)		2.5	2.5	0.0	1.000
SGLT2-inhibitors (%)		11.0	8.0	12.0	0.273
GLP-1 RAs (%)		7.0	7.0	0.0	0.545
T2DM (%)		17.5	17.7	16.7	0.903
Metabolic Syndrome (%)		23.9	23.3	27.3	0.687
Microalbuminuria (%)		15.5	12.9	26.1	0.118
Creatinine (mg/dL)		0.87 ± 0.25	0.88 ± 0.25	0.86 ± 0.25	0.793
eGFR (mL/min)		86.2 ± 19.8	86.9 ± 20.0	81.9 ± 18.3	0.249
ABPM					
Daily mean	SBP (mmHg)	131.0 ± 16.0	130.6 ± 16.7	132.6 ± 13.5	0.603
	DBP (mmHg)	76.8 ± 9.8	77.7 ± 10.0	73.3 ± 8.0	0.064
	HR (bpm)	74.9 ± 9.8	75.4 ± 8.7	72.9 ± 13.0	0.287
	HRV (bpm)	11.8 ± 3.7	11.8 ± 3.5	11.8 ± 4.5	0.985
	PP (mmHg)	53.9 ± 11.6	52.9 ± 10.7	57.6 ± 14.1	0.096
Daytime mean	SBP (mmHg)	131.0 ± 16.0	136.4 ± 17.1	136.9 ± 13.7	0.916
	DBP (mmHg)	81.0 ± 10.4	82.3 ± 10.6	76.5 ± 8.5	0.023
	HR (bpm)	78.1 ± 10.5	78.7 ± 9.8	76.1 ± 13.0	0.308
	HRV (bpm)	11.6 ± 3.8	11.5 ± 3.5	11.9 ± 4.7	0.722
	PP (mmHg)	55.5 ± 11.0	54.2 ± 11.1	60.4 ± 9.6	0.021
Nighttime mean	SBP (mmHg)	131.0 ± 16.0	115.7 ± 18.5	122.1 ± 16.1	0.153
	DBP (mmHg)	65.9 ± 10.3	66.1 ± 10.7	65.2 ± 8.5	0.735
	HR (bpm)	66.9 ± 10.1	67.4 ± 8.6	65.0 ± 14.6	0.338
	HRV (bpm)	5.5 ± 2.4	5.8 ± 2.4	4.6 ± 2.2	0.050
	PP (mmHg)	51.2 ± 11.8	49.7 ± 11.5	56.9 ± 11.4	0.012
Dipping (%)	Reverse	1.0	1.3	0.0	0.152
	Reduced	28.3	23.1	47.6	
	Normal	46.5	48.7	38.1	
	Extreme	24.2	26.9	14.3	
AASI		0.47 ± 0.15	0.45 ± 0.15	0.53 ± 0.17	0.052

*ACEi* angiotensin converting enzyme inhibitors, *AASI* ambulatory arterial stiffness index, *ARBs* angiotensin II receptor blockers, *ARR* aldosterone-to-renin ratio, *BMI* body mass index, *CCB* calcium channel blocker, *cIMT* carotid intima-media thickness, *CVD* cardiovascular disease, *DBP* diastolic blood pressure, *dU* daily urinary, *eGFR* estimated glomerular filtration rate, *FH* family history, *GLP-1 RA* glucagon-like peptide-1 receptor agonists, *HR* heart rate, *HRV* heart rate variability, *MRA* mineralocorticoid receptor antagonist, *NFAI* nonfunctioning adrenal incidentaloma, *PP* pulse pressure, *SBP* systolic blood pressure, *SGLT2i* sodium–glucose cotransporter-2 inhibitors, *T2DM* type 2 diabetes mellitus

**Table 2:** Arterial stiffness and carotid Doppler ultrasound data

Arterial Stiffness				
Variables/Parameters	Total	Controls	NFAI	p-value
Sphygmocor Reference Age (years)	68.47 ± 16.13	67.88 ± 16.55	72.42 ± 12.59	0.200
SBP (mmHg)	139.4 ± 20.0	138.9 ± 20.4	142.4 ± 17.1	0.424
DBP (mmHg)	79.1 ± 12.0	79.5 ± 12.3	76.2 ± 9.8	0.199
PPP (mmHg)	60.3 ± 15.2	59.4 ± 14.9	66.2 ± 16.1	0.038
MAP (mmHg)	100.1 ± 14.3	100.2 ± 14.7	98.9 ± 11.7	0.668
Central SBP (mmHg)	127.0 ± 17.3	126.6 ± 17.6	129.7 ± 15.3	0.402
Central DBP (mmHg)	80.4 ± 12.4	80.8 ± 12.8	77.7 ± 10.0	0.254
Central PP (mmHg)	46.6 ± 13.1	45.8 ± 12.6	52.0 ± 15.0	0.027
Heart Rate (bpm)	74.5 ± 12.6	75.0 ± 12.9	71.1 ± 10.3	0.149
Period (ms)	827.07 ± 132.76	822.03 ± 134.78	860.32 ± 115.50	0.180
Ejection Duration (ms)	318.40 ± 29.02	317.57 ± 28.61	323.84 ± 31.67	0.315
Ejection Duration (%)	0.39 ± 0.05	0.39 ± 0.05	0.38 ± 0.04	0.199
Aortic T2 (ms)	221.08 ± 19.92	220.48 ± 20.21	225.04 ± 17.75	0.287
P1 Height (mmHg)	34.90 ± 9.01	34.35 ± 8.83	38.52 ± 9.54	0.031
Augmented Pressure (mmHg)	16.41 ± 9.69	15.95 ± 9.41	19.48 ± 11.10	0.089
Aortic AIx AP/PP (%)	0.33 ± 0.14	0.33 ± 0.14	0.36 ± 0.13	0.359
Aortic AIx P2/P1 (%)	1.34 ± 0.13	1.33 ± 0.14	1.35 ± 0.12	0.682
Aortic AIx@HR75 (%)	0.33 ± 0.12	0.33 ± 0.13	0.34 ± 0.11	0.862
Buckberg SEVR (%)	1.27 ± 0.24	1.27 ± 0.25	1.27 ± 0.19	0.904
PTI Systole (mmHg·s/min)	2691 ± 544	2699 ± 562	2632 ± 411	0.565
PTI Diastole (mmHg·s/min)	3313 ± 487	3315 ± 499	3302 ± 408	0.907
End Systolic Pressure (mmHg)	114.9 ± 15.8	114.7 ± 16.1	116.1 ± 13.9	0.679
MAP Systole (mmHg)	114.1 ± 15.2	114.0 ± 15.6	115.4 ± 12.4	0.655
MAP Diastole (mmHg)	91.1 ± 14.1	91.4 ± 14.4	89.0 ± 12.0	0.424
PP Amplification (%)	1.31 ± 0.13	1.31 ± 0.12	1.29 ± 0.13	0.576
Forward Pulse Height (mmHg)	33.93 ± 9.13	33.26 ± 8.75	38.32 ± 10.49	0.009
Reflected Pulse Height (mmHg)	20.43 ± 6.22	20.10 ± 6.10	22.60 ± 6.70	0.061
Reflection magnitude (%)	0.61 ± 0.12	0.61 ± 0.12	0.60 ± 0.11	0.582
Pulse Transit Time (ms)	60.87 ± 12.57	60.83 ± 12.13	61.08 ± 15.39	0.928
PWV (m/s)	8.77 ± 1.77	8.70 ± 1.70	9.23 ± 2.18	0.163
Carotid Doppler ultrasound				
Mean left cIMT (µm)	769.14 ± 177.80	769.23 ± 185.56	768.56 ± 122.26	0.986
SD left cIMT (µm)	52.52 ± 45.87	52.55 ± 46.64	52.32 ± 41.65	0.982
Max left cIMT (µm)	829.89 ± 195.40	824.36 ± 192.46	864.20 ± 213.74	0.346
Mean left diameter (mm)	7.05 ± 1.22	7.08 ± 1.17	6.86 ± 1.52	0.396
SD left diameter (mm)	0.49 ± 0.70	0.46 ± 0.66	0.68 ± 0.90	0.139
Max left diameter (mm)	7.42 ± 1.09	7.41 ± 1.09	7.48 ± 1.09	0.764
Mean right cIMT (µm)	720.57 ± 139.83	718.57 ± 145.74	733.33 ± 95.18	0.632
SD right cIMT (µm)	41.86 ± 38.89	42.48 ± 40.22	37.96 ± 29.46	0.598
Max right cIMT (µm)	780.96 ± 158.97	779.33 ± 166.64	791.00 ± 101.43	0.734
Mean right diameter (mm)	7.13 ± 1.22	7.05 ± 1.21	7.62 ± 1.21	0.034
SD right diameter (mm)	0.54 ± 0.72	0.52 ± 0.72	0.66 ± 0.78	0.406
Max right diameter (mm)	7.51 ± 1.09	7.41 ± 1.08	8.11 ± 0.94	0.003
cIMT≥0.9 mm (%)	36.9	34.4	52.0	0.091
Presence of carotid stenosis (%)	14.8	13.3	25.0	0.133

*AIx* augmentation index, *AP* augmentation pressure, *AIx@HR75* augmentation index normalized for 75bpm, *cIMT* carotid intima-media thickness, *DBP* diastolic blood pressure, *MAP* mean arterial pressure, *Max* maximum measure, *NFAI* nonfunctioning adrenal incidentaloma, *PP* pulse pressure, *PPP* peripheral pulse pressure, *PTI* pressure time integral, *PWA* Pulse Wave Analysis, *PWV* Pulse Wave Velocity, *SBP* systolic blood pressure, *SD* standard deviation

**Table 3:** Multiple linear regression of the association between NFAI/cardiometabolic confounders and outcomes of pulse wave analysis (augmentation index)

Covariates	AIx HR75				AIx AP/PP				AIx P2/P1			
	EC	95% CI		p-value	EC	95% CI		p-value	EC	95% CI		p-value
NFAI	1.073	1.004	1.147	0.039	1.133	1.043	1.232	0.004	1.097	1.016	1.185	0.019
Male sex	0.907	0.856	0.961	0.001	0.917	0.854	0.984	0.017	0.922	0.863	0.984	0.015
BMI (Kg/m^2^)	0.998	0.991	1.005	0.491	0.992	0.984	1.000	0.062	0.994	0.987	1.002	0.140
SBP (mmHg)	1.001	0.999	1.002	0.328	1.000	0.998	1.002	0.924	0.999	0.997	1.001	0.218
T2DM	0.959	0.881	1.044	0.328	0.978	0.879	1.088	0.676	0.966	0.876	1.066	0.489
eGFR (mL/min)	1.001	0.999	1.003	0.390	1.000	0.998	1.002	0.803	1.000	0.998	1.002	0.936
dU-normetanephrine (μg/day)	1.000	0.999	1.000	0.123	1.000	0.999	1.000	0.380	1.000	0.999	1.000	0.417
No of antihypertensive drugs	1.012	0.986	1.039	0.364	1.021	0.988	1.055	0.210	1.015	0.985	1.046	0.330

*AIx* augmentation index, *AIx AP/PP* AIx calculated as the ratio of Augmentation Pressure (AP) to Pulse Pressure (PP), *AIx HR75* AIx normalized for a heart rate of 75 bpm, *AIx P2/P1* AIx calculated as the ratio of the amplitude of the second systolic peak (P2) to the first systolic peak (P1), *BMI* body mass index, *CI* confidence interval, *EC* exponentiated β-coefficient, *eGFR* estimated glomerular filtration rate, *NFAI* nonfunctioning adrenal incidentaloma, *SBP* systolic blood pressure, *T2DM* type 2 diabetes mellitus, *dU* daily urinary

However, patients with NFAI had significantly lower total cholesterol and LDL cholesterol levels compared to controls (p = 0.034 and p = 0.028, respectively), likely reflecting a higher use of lipid-lowering agents in this subgroup.

As shown in Table 1, ABPM revealed specific hemodynamic differences. NFAI patients exhibited lower daytime diastolic blood pressure (DBP) (76.5 ± 8.5 mmHg vs 82.3 ± 10.6 mmHg, p = 0.023) and higher daytime PP (60.4 ± 9.6 mmHg vs 54.2 ± 11.1 mmHg, p = 0.021), compared to controls. Nighttime PP was also elevated in the NFAI group (56.9 ± 11.4 mmHg vs 49.7 ± 11.5 mmHg, p = 0.012), along with a trend toward reduced nighttime HRV (p = 0.050). Although plasma normetanephrine levels were not significantly different, their numerical increase, in conjunction with ABPM findings, may reflect subtle sympathetic activation in NFAI patients.

The distribution of antihypertensive and cardiometabolic treatments was overall comparable between NFAI patients and controls. The use of ACE inhibitors, angiotensin II receptor blockers, calcium channel blockers, thiazide (-like) diuretics, mineralocorticoid receptor antagonists, β-blockers, SGLT2 inhibitors, and GLP-1 receptor agonists did not differ significantly between groups. The only exception was α-blocker therapy, which was more frequently prescribed in NFAI patients (17.4% vs. 4.2% in controls, *p* = 0.038; Table 1).

Table 2 illustrates arterial stiffness profiles. Although PWV did not differ significantly between groups (*p* = 0.163), NFAI patients showed higher peripheral PP (PPP: 66.2 ± 16.1 vs. 59.4 ± 14.9 mmHg; p = 0.038), central PP (52.0 ± 15.0 vs. 45.8 ± 12.6 mmHg; p = 0.027), and forward pulse height (38.3 ± 10.5 vs. 33.3 ± 8.8 mmHg; p = 0.009), all indicative of reduced arterial compliance and increased wave reflection.

Carotid Doppler ultrasound findings are summarized in Table 2. Although the difference in the proportion of subjects with cIMT ≥0.9 mm did not reach statistical significance (52.0% in NFAI vs. 34.4% in controls; p = 0.091), NFAI patients exhibited significantly larger right carotid diameters, both mean (7.62 ± 1.21 vs. 7.05 ± 1.21 mm; p = 0.034) and maximal (8.11 ± 0.94 vs. 7.41 ± 1.08 mm; p = 0.003), suggesting structural vascular remodeling.

### Correlation analysis of clinical, biochemical, and vascular variables/parameters

**Fig. 2:**
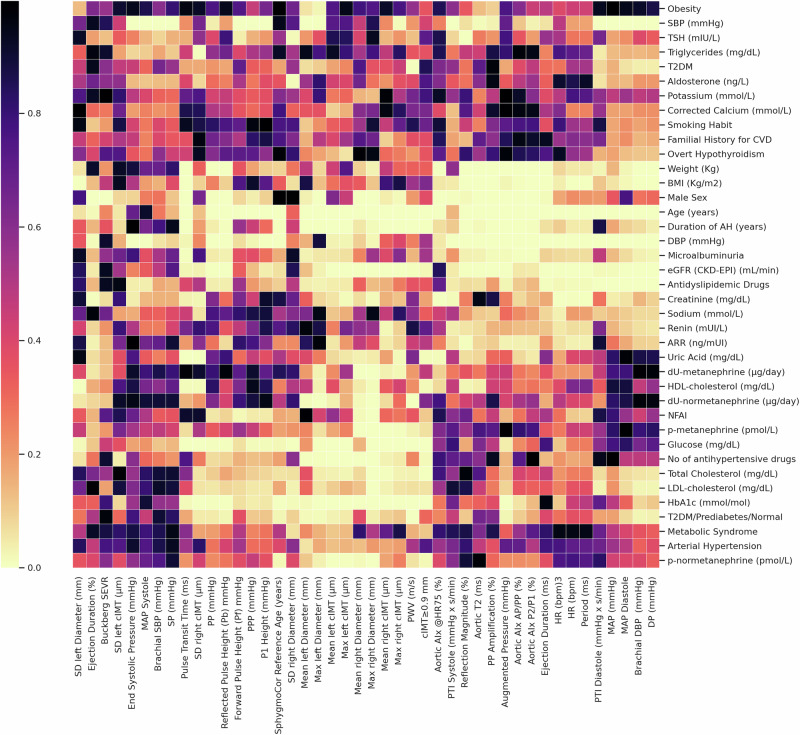
Clustered heatmap of p-values from pairwise correlation analyses between clinical/biochemical parameters (rows) and vascular/imaging-derived variables (columns). The matrix includes continuous variables tested with Spearman’s rank correlation and categorical variables analyzed using Kruskal-Wallis tests. Hierarchical clustering was applied to both rows and columns, but dendrograms are not shown for clarity. The color scale reflects the statistical significance of each association, with yellow indicating lower p values (stronger associations) and black indicating non-significant results (magma_r colormap). AH arterial hypertension, AIx augmentation index, ARR aldosterone-to-renin ratio, BMI body mass index, cIMT carotid intima-media thickness, CVD cardiovascular disease, CKD-EPI Chronic Kidney Disease Epidemiology Collaboration, DBP diastolic blood pressure, DP diastolic pressure, HDL high-density lipoprotein cholesterol, HR heart rate, LDL low-density lipoprotein cholesterol, MAP mean arterial pressure, Max maximum measure, NFAI nonfunctioning adrenal incidentaloma, P1 first systolic peak pressure, PP pulse pressure, PPP peripheral pulse pressure, PTI pressure time integral, PWV Pulse Wave Velocity, SBP systolic blood pressure, SD standard deviation, SP systolic pressure, T2DM type 2 diabetes mellitus

The heatmap analysis revealed distinct clusters of significant associations between clinical, biochemical, and anthropometric variables and indices of arterial stiffness and atherosclerosis (Fig. 2). Two main clusters emerged. The first included age, duration of arterial hypertension, weight, BMI, DBP, microalbuminuria, eGFR, serum creatinine, and aldosterone-to-renin ratio, all strongly associated with AIx and wave reflection parameters. This cluster reflects a shared vascular aging phenotype characterized by renal impairment, altered hemodynamics, and early arterial remodeling.

The second cluster grouped age, hypertension duration, markers of glucose and lipid metabolism (e.g., T2DM, prediabetes, triglycerides, HDL cholesterol), which were significantly correlated with cIMT and PWV, consistent with a metabolic–vascular aging profile.

Additional associations were noted between BMI, body weight, HbA1c, and arterial stiffness parameters, including AIx, PWV, and SBP amplification, suggesting that metabolic dysregulation contributes to vascular dysfunction.

Markers of sympathetic tone, such as plasma and urinary metanephrines, were associated with PWV and several indices of vascular remodeling, suggesting a potential link between low-grade sympathetic activation and cardiovascular risk.

Moreover, reduced eGFR and elevated uric acid were associated with increased central PP and AIx, reinforcing the role of renal dysfunction in arterial aging.

Notably, NFAI itself formed a distinct cluster with AIx, central systolic pressure, PWV, and cIMT, supporting its association with early vascular remodeling and an adverse cardiometabolic profile, even in the absence of overt hormonal hypersecretion.

### Multivariable analysis

Multivariable linear and logistic regression analyses were performed to explore the independent associations between NFAI and vascular parameters.

As reported in Table 3, NFAI was associated with increased:

- AIx@HR75 (EC 1.073, 95% CI 1.004–1.147; p = 0.039), after adjusting for male sex (EC 0.907, 95% CI 0.856-0.961; p = 0.001), BMI, T2DM, eGFR, number of antihypertensive drugs, SBP, and dU-normetanephrine;

- AIx AP/PP (EC 1.133, 95% CI 1.043–1.232; p = 0.004), after adjusting again for male sex (EC 0.917, 95% CI 0.854–0.984; p = 0.017), BMI, T2DM, eGFR, number of antihypertensive drugs, SBP, and dU-normetanephrine;

- AIx P2/P1 (EC 1.097, 95% CI 1.016–1.185; p = 0.019), also adjusting for male sex (EC 0.922, 95% CI 0.863–0.984, p = 0.015), BMI, T2DM, eGFR, number of antihypertensive drugs, SBP, and dU-normetanephrine.

As shown in Table 4, PWV was significantly higher in NFAI patients (EC 2.426, 95% CI 1.038–5.674; p = 0.041), independently of male sex, BMI, SBP (EC 1.032, 95% CI 1.011–1.053; p = 0.003), T2DM, eGFR, and number of antihypertensive drugs.

**Table 4:** Multiple linear regression and multivariable logistic regression analysis of the association between NFAI and PWV/cIMT ≥ 0.9 mm

PWV	EC	95% CI		p-value
NFAI	2.426	1.038	5.674	0.041
Male sex	1.592	0.796	3.183	0.186
BMI (Kg/m^2^)	0.972	0.903	1.047	0.455
SBP (mmHg)	1.032	1.011	1.053	0.003
T2DM	1.964	0.717	5.382	0.187
eGFR (mL/min)	0.985	0.967	1.004	0.115
No of antihypertensive drugs	1.290	0.969	1.718	0.081

cIMT ≥0.9 mm	OR	95% CI		p-value
NFAI	3.312	1.011	10.855	0.048
Age	1.033	0.993	1.075	0.103
Male sex	1.655	0.643	4.257	0.296
BMI (Kg/m^2^)	0.912	0.818	1.017	0.099
SBP (mmHg)	1.005	0.973	1.038	0.760
DBP (mmHg)	1.026	0.968	1.087	0.385
T2DM	3.219	0.933	11.103	0.064
eGFR (mL/min)	0.990	0.965	1.016	0.446
No of antihypertensive drugs	1.628	1.146	2.313	0.007

*BMI* body mass index, *CI* confidence interval, *cIMT* carotid intima-media thickness, *DBP* diastolic blood pressure, *EC* exponentiated β-coefficient, *eGFR* estimated glomerular filtration rate, *NFAI* nonfunctioning adrenal incidentaloma, *PWV* pulse wave velocity, *SBP* systolic blood pressure

No age adjustment was applied to AIx and PWV, as age is inherently part of their assessment.

Finally, multivariable logistic regression (Table 4) demonstrated that NFAI was independently associated with increased cIMT ( ≥ 0.9 mm) (OR 3.312, 95% CI 1.011–10.855; p = 0.048), after adjusting for age, sex, T2DM, BMI, number of antihypertensive drugs (OR 1.628, 95% CI 1.146–2.313; p = 0.007), eGFR, SBP, and DBP.

## Discussion

To our knowledge, this is the first study to comprehensively evaluate vascular health in patients with NFAI, compared to unselected controls, using gold standard for arterial stiffness (SphygmoCor Xcel) and advanced carotid Doppler ultrasound (QIMT and plaque detection). Despite their presumed endocrine inactivity, our findings consistently demonstrate that NFAIs are associated with subclinical alterations in vascular structure and function.

Adrenal tumors are commonly discovered on cross-sectional abdominal imaging in up to 10% of patients [1, 27]. Previous studies conducted in recent years shows the negative effects of NFAI, relating it to increased BP levels [5, 8, 28], insulin resistance [10, 11, 14, 29, 30], rate of dyslipidaemia, diabetes, obesity [4, 9, 31], echocardiographic alterations [12, 13, 32, 33], and CV events [7]. A recent meta-analysis [34] confirmed that NFAIs are associated with arterial hypertension, prediabetes/T2DM and metabolic syndrome, but not with only T2DM, dyslipidemia and CV events. Another study did not find association between NFAI and cardiovascular mortality [35].

Data on relationship between NFAI and arterial stiffness derived from few studies performed with non-gold standard devices for the determination of PWA and PWV (8,10).

In this study, from a metabolic standpoint, NFAI patients displayed significantly lower levels of total and LDL-cholesterol compared to controls, a finding that may reflect a higher rate of lipid-lowering treatment in this subgroup. No differences emerged in glucose levels or prevalence of diabetes, although a numerical trend toward lower HbA1c was observed in the NFAI group.

Regarding BP, office measurements did not differ between groups, but ABPM revealed lower daytime DBP and higher PP in NFAI patients, both during daytime and nighttime periods. These findings suggest increased arterial stiffness, which was further corroborated by PWA.

Indeed, among parameters of central hemodynamics, patients with NFAI showed significantly higher central PP, forward pulse height, and P1 amplitude, all indicative of reduced aortic compliance and enhanced wave reflection. While PWV did not reach statistical significance in univariate comparison, multivariable regression analysis identified NFAI as an independent predictor of higher PWV, even after adjusting for other cardiometabolic confounders. Similarly, NFAI was independently associated with higher values of AIx, reinforcing the hypothesis of early functional vascular changes in these patients.

Carotid Doppler ultrasound further supported this vascular phenotype. Although mean cIMT did not differ between groups, NFAI patients exhibited significantly larger right carotid diameters (both mean and maximal). Of note, the left carotid diameter was also numerically higher in NFAI patients compared to controls, although the difference did not reach statistical significance. This pattern may suggest a trend toward bilateral vascular remodeling, with significance observed only on the right side possibly due to variability or sample size limitations. Alternatively, subtle asymmetry in vascular remodeling could reflect anatomical or hemodynamic differences (e.g., dominance of the right carotid pathway or local flow dynamics).

Moreover, the prevalence of individuals with cIMT ≥0.9 mm, a surrogate marker of subclinical atherosclerosis, was numerically higher in NFAI subjects and was independently predicted by the presence of NFAI in logistic regression analysis.

Taken together, these findings highlight a consistent pattern: NFAI is associated with subtle but measurable alterations in vascular structure and function, including increased arterial stiffness, abnormal pressure wave dynamics, and early atherosclerotic remodeling. These changes occur independently of overt endocrine secretion, suggesting that the presence of NFAI may reflect broader systemic alterations in vascular aging or cardiometabolic homeostasis.

The pathophysiological underpinnings of this association remain to be fully elucidated. Potential mechanisms may include low-grade cortisol autonomy below classical thresholds [36, 37], increased local vascular sensitivity to glucocorticoids, undetectable corticosteroid secretion with routine exams or altered corticosteroid profile [6, 38], alteration of the sympathetic tone [39, 40], or shared developmental pathways (genetic background) between adrenal and vascular tissues. Alternatively, NFAI may act as a marker of occult systemic dysregulation (i.e., insulin resistance) rather than a causal factor per se [41].

The findings of this study have important implications for the management of NFAI patients in the future. Having an increased risk of arterial stiffness and atherosclerosis, NFAI patients should undergo regular cardiometabolic risk assessment, including PWA, PWV, and carotid artery assessment. Early identification of vascular changes may allow for timely intervention, potentially reducing the risk of CV events.

While adrenalectomy remains controversial in hormonally inactive lesions, a more proactive approach may be justified in selected patients with adverse vascular phenotypes. Future studies should clarify whether early treatment improves outcomes and explore mechanisms linking NFAI with vascular dysfunction.

This study has several strengths, including standardized vascular assessments performed by a single blinded operator (M.P.C.), a relatively large and clinically relevant control group (patients with suspicion of secondary hypertension with CV risk factors, as arterial hypertension, T2DM, dyslipidemia, and obesity), and robust multivariable analyses. Limitations include its cross-sectional design and limited NFAI sample size.

Longitudinal studies are needed to confirm causality and evaluate whether early intervention in NFAI patients can alter cardiovascular risk trajectories. Future research is still needed to explore the underlying mechanisms through which NFAI can lead to increased arterial stiffness and endothelial dysfunction. Understanding these mechanisms may lead to the development of targeted therapeutic strategies to improve vascular and metabolic health in NFAI patients.
